# Mental health in the moment: protocol for an accelerated cohort measurement burst study of adolescent mental health

**DOI:** 10.1136/bmjopen-2025-110190

**Published:** 2025-11-12

**Authors:** Aja Murray, Luke Power, Dejla Hoxha, Tong Xie, Helen Wright, Lorna Caddick, Katie Dryburgh, Clara Sanchez-Izquierdo, Daria Melashenko, Alex Crocker

**Affiliations:** 1 Department of Psychology, University of Edinburgh, Edinburgh, UK; 2 Faculty of Psychology, Beijing Normal University, Beijing, China; 3 Division of Psychiatry, University of Edinburgh, Edinburgh, UK; 4 University of Edinburgh, Edinburgh, UK

**Keywords:** MENTAL HEALTH, Adolescent, Depression & mood disorders, Anxiety disorders

## Abstract

**Abstract:**

**Introduction:**

Adolescence is a key period of development for mental health; however, little is known about how (cumulative) daily life experiences impact long-term mental health development in this period, and vice versa. ‘Mental health in the moment’ (MHIM) is an accelerated cohort measurement burst study designed to illuminate these links.

**Methods and analysis:**

The current protocol describes the rationale and design for MHIM, which aims to recruit and follow up approximately 500 adolescents across five age cohorts (in secondary school years S1–S5, aged 11–16 at baseline) and follow them over a 5-year data collection period. Data collection will include online surveys and ecological momentary assessments bursts every 6 months, annual caregiver surveys, the collection of stress biomarker data at three key measurement points and continuous radar-based sleep measurement for a subsample of participants. The study is informed by a young person advisory group input throughout its lifecycle. Data will be analysed using techniques such as dynamic structural equation modelling. The study can provide insights into mental health development from a multitimeframe developmental perspective, including insights into ‘daily life’ intervention targets for improving adolescent mental health.

**Ethics and dissemination:**

The study received ethical approval from the philosophy, psychology and language science ethics committee at the University of Edinburgh (404-2425/3) and the findings will be published in a series of peer-reviewed publications.

**STRENGTHS AND LIMITATIONS OF THIS STUDY**

- The measurement burst design can link day-to-day experiences to long-term development.
- The accelerated cohort design increases the developmental span but relies on the assumption of cohort invariance.
- Capturing data during the school day can be challenging because of smartphone use policies.
- Participant retention over a 5-year period will be challenging.

## Background and rationale

Adolescence is a peak period of vulnerability for the onset and escalation of mental health issues, especially internalising problems.^1 2^ Many important influences on adolescent mental health are those that play out on a day-to-day basis, such as sleep, social interactions and stressors, as well as the cognitive, emotional and behavioural reactions they evoke.^3^,^7^ Importantly, these factors can show reciprocal relations with mental health outcomes.^8^,^10^ However, how cumulative daily life experiences in the formative period of adolescence are linked to long-term mental health development is little understood. The current protocol describes a planned multitimeframe longitudinal study that can help illuminate these links. In doing so, it seeks to offer new insights into intervention targets that lie within the daily life experiences of adolescents.

There is no universally accepted definition of adolescence; however, there is a strong consensus that it represents a critical period for mental health development.^2 11^ The period is characterised by a range of biological (eg, hormonal), social and psychological changes with implications for mental health. For example, adolescents must navigate challenges such as identity formation,^12^ an intensification of the importance of peer relationships,^13^ the emergence of romantic involvement,^3^ circadian rhythm shifts^14 15^; and heightened emotional and behavioural regulation challenges.^16^,^18^ These occur in the context of increased expectations of independence and diminishing parental monitoring.^19^ Further, increased plasticity of the brain and systems such as the hypothalamic–pituitary–adrenal axis creates pathways by which experiences during this time can have long-term significance for mental health trajectories.^6^

These considerations make a developmental perspective essential for understanding adolescent mental health,^2 12^ and correspondingly, important advances in our understanding of mental health in this period have come from longitudinal study designs.^20^,^22^ However, the presence of longer-term changes occurring over months and years with connections to experiences occurring over minutes, hours and days^23^,^28^ necessitates studies capturing data over multiple timescales.^29^ Few studies have attempted to link ‘developmental’ (months, years) and ‘momentary’ (minutes, hours, days) time in this way, tending to focus on one or the other timeframe.^30^

Measurement burst designs^31^ can help bridge this gap and illuminate the links between daily life experiences and long-term mental health development. Measurement burst designs involve repeated ‘bursts’ of ecological momentary assessment (EMA) data collections. In each EMA burst, short surveys can be used to gather intensive in-the-moment data about experiences as they play out in the course of participants’ daily lives.^32^ This is commonly implemented via smartphone-based surveys administered several times a day over the course of a number of days or weeks.^33 34^ EMA has offered numerous insights into the mental health of young people,^35^ such as how specific affective dynamics (eg, emotional lability and inertia) are related to different mental health outcomes.^36^ Repeating the EMA at regular intervals in a burst design facilitates the examination of developmental change and/or the reciprocal relations between daily life experiences and longer-term change.^37^ However, very few EMA measurement burst studies have been conducted to date and only a handful focus on mental health,^38 39^ with studies in adolescence particularly lacking.^40^

### The present study

Given the need for evidence on the reciprocal relations between daily life experiences and long-term mental health development in the critical period of adolescence, we here outline our plans for a multitimeframe study that combines traditional caregiver and young person surveys, EMA bursts, passive sleep sensing and biosampling. The study will use an accelerated cohort design^41^ to capture developmental data spanning ages 11–20 over a 5-year data collection period. A multi-informant approach including both caregiver and adolescent self-reports will be taken in line with the fact that young people may show different behaviours in different contexts and with different informants.^42 43^ Caregivers and adolescents will complete online surveys and adolescents will additionally complete smartphone-based EMA surveys and provide biosamples. A subsample will also complete a radar-based ambient sleep measurement protocol. Overarching research questions will include:

- How do cumulative daily life experiences such as social experiences and daily stressors impact on mental health development across adolescence?
- How do changes in mental health status (eg, increases or decreases in depression) impact on daily life experiences (eg, emotions and their regulation)?
- How do changes in factors such as emotion regulation in daily life and sleep over the course of adolescence impact on mental health?
- What are the developmental differences across adolescence in the daily life influences on, and manifestation of, mental health issues?

## Methods

### Patient and public involvement

The mental health in the moment young person advisory groups (MHIM-YPAG), alongside other YPAGs, were consulted on the design of the study and will provide input throughout the lifecycle of the project, including the design of the study, interpretation of results and dissemination of findings. Thus far, YPAGs have provided advice on which mental health influences and outcomes to include in the study, the engagement strategies^44^ and the codesign procedure.^45^ Full details of the planned coproduction are provided in a dedicated publication^45^; however, in brief, the process will involve adolescents aged 11–18 engaged via a series of YPAGs across >35 sessions over the project. These are divided into a ‘predata collection’ phase and a ‘data collection phase’. Sessions will focus on topics such as ‘mental health concepts’, ‘recruitment strategy and materials’, ‘mental health instruments and survey design feedback’, ‘hair sampling and EMA and sleep measurement’, ‘data chat and dissemination’, ‘training needs’, ‘training’, ‘informal team building’ and ‘output’. Given the emphasis on young people’s input, the design described in this protocol is subject to change based on the views of the MHIM-YPAG.

### Overview

**Figure 1:**
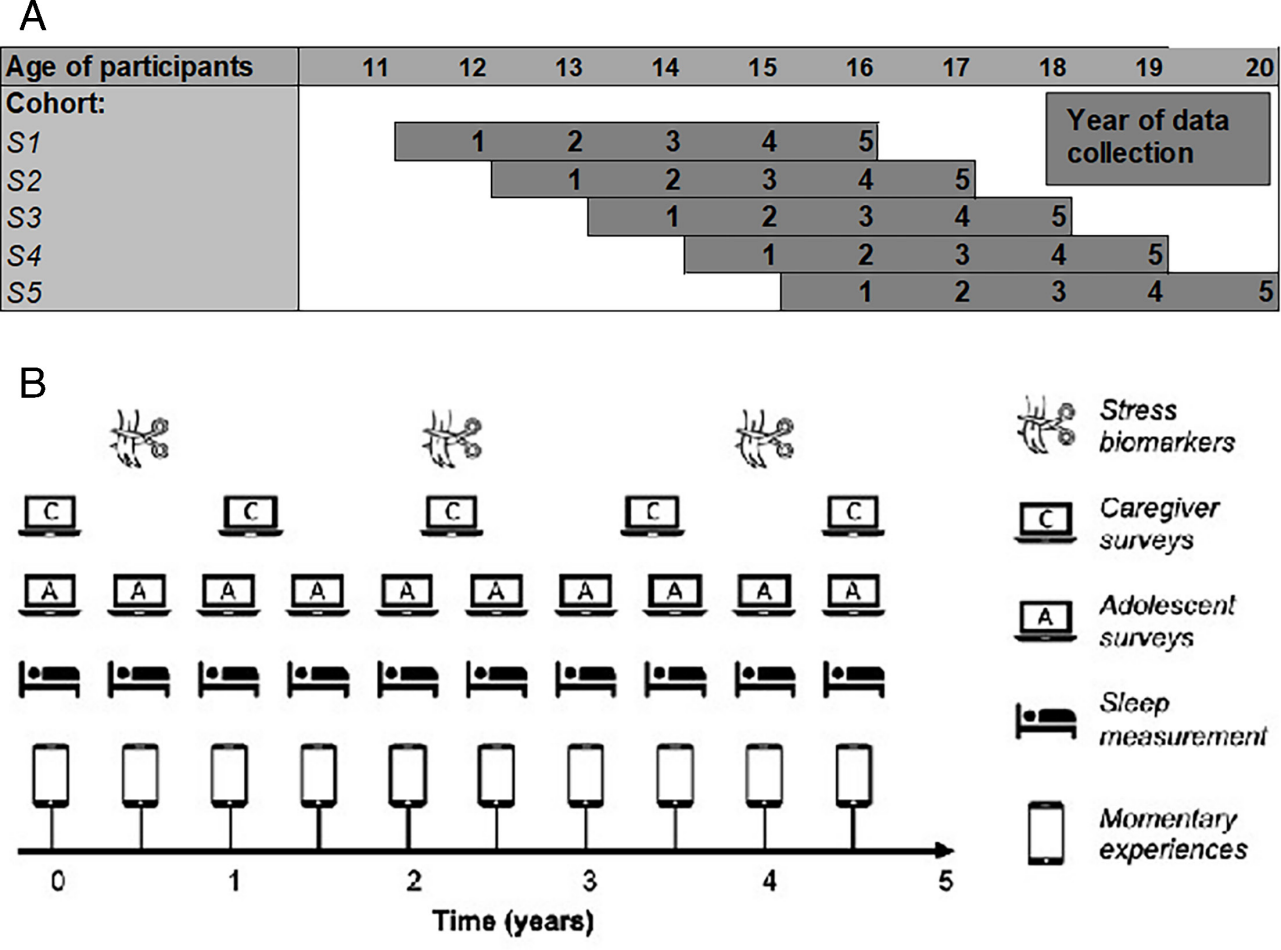
(A) The accelerated cohort design with 5 age cohorts followed over a 5-year period. The cohorts will be recruited by school year (S1–S5), which corresponds approximately to age 11/12 to 15/16. (B) The data collection schedule for mental health in the moment.

The study will use a measurement burst accelerated cohort design.^31 46^ Specifically, every 6 months over a 5-year period, adolescents will complete an EMA burst and an online survey. At baseline (anticipated beginning in November 2025), five cohorts of approximately n=150 will be recruited from schools in the UK (see figure 1) to facilitate developmental data spanning ages 11–20 in 5 years of data collection (ending in December 2030). We aim to keep the intervals between data collections similar across young people but will test and, if necessary, later statistically adjust for any effects of individual differences in intermeasurement time intervals at the analysis stage.

Hair samples will be collected at the end of selected EMA/online survey bursts to provide biomarkers of stress concurrent with EMA periods.^47 48^ Participants who are unable or who prefer not to provide a hair sample will be offered the option to provide a nail sample. This will be used to impute hair cortisol and cortisone values for these participants. A pilot study, which will analyse the correlation between hair and nail sample biomarker values, will be used to validate this method.

Participants will be offered compensation for taking part, scaled to their level of engagement across the different components (surveys, EMA, biosampling and sleep measurement).

### Participants

Our baseline sample will be 750 adolescents from schools in Scotland (oversampling at baseline to help ensure adequate numbers remain at follow-up waves to support longitudinal analyses). Though definitions of adolescence vary, there is general consensus that ages 11–19 fall within adolescence within Western contexts^49^ and this age range will thus be the focus of the present study. The recruitment and data collection will be implemented via local schools, reflecting the fact that the school environment is of considerable importance with respect to fostering the development of good mental health and potentially for the delivery of preventive interventions.^50^ Sex and gender differences have been shown to be important in mental health^51 52^; therefore, we will aim to ensure sex and gender diversity in the sample.

At baseline, n=150 participants from each of 5 groups defined by school year will be recruited: secondary school years 1, 2, 3, 4 and 5 at the time of recruitment (approximately corresponding to an age range of 11–16). If necessary, refreshment samples will be considered to counteract dropout.^53 54^ Participants will be approached via their schools, with the research team sharing information about the study via posters and presentations and links for signing up shared by schools with parents/caregivers.

To help maximise sample diversity and minimise recruitment bias, our goal is to work with schools situated in both rural and urban areas with varying levels of socioeconomic deprivation. We will take measures to help ensure that our recruitment materials and strategies are inclusive, such as producing poster adverts depicting young people of different genders and ethnicities and seeking advice on our strategy and materials from our YPAG.

### Measures

#### Measures overview

There are multiple measurement components: EMA, young person intake survey, caregiver intake survey, young person six-monthly survey, parental annual survey, ambient sleep measurement, biosampling (and associated biosampling survey).

The *young person and caregiver intake surveys* will also allow us to gather sociodemographic information that will facilitate assessment of the representativeness of the sample and to potentially derive survey and attrition weights,^55^ as well as substantive information about participant mental health and its influences. The caregiver and young person intake surveys will include complementary information as well as a subset of the same measures from the parent and young person’s perspective to provide a multi-informant perspective. Tables1^3^ summarise the measures. However, measures remain subject to change based on input from the YPAG over the course of the study and may be supplemented with others for future ‘add-on’ studies. Some measures will be administered only on an annual or less frequent basis, and others will be administered only up to a given point (eg, when the highest level of pubertal development is reached).

**Table 1:** Summary of young person survey measures

Measure	Frequency
Positive and negative mental health outcomes	
Revised Children’s Anxiety and Depression Scale (RCADS)^91 92^	RCADS-25 administered annually, RCADS-11 administered every 6 months
Adapted and abbreviated version of the Youth Suicidal Ideation Screen 3^93^	Every 6 months
WHO Wellbeing Scale (WHO-5) (WHO, 1998)^94^	Every 6 months
Youth Quality of Life Short Form^95^	Annual
Abbreviated Problem Behavior Frequency Scale^96^	Annual
Substance use indicators adapted from *Understanding Society*	Annual
Abbreviated version of Rosenberg Self-Esteem Scale^97^	Every 6 months
Abbreviated version of the Perceived Stress Scale^98^	Every 6 months
Mental health influences	
Pubertal Development Scale^15^	Every 6 months until highest level reached
Social Behaviour Questionnaire-Attention Deficit Hyperactivity Disorder (SBQ-ADHD) subscale^60 99^	Every 6 months
Abbreviated version of the Adolescent Sleep Hygiene Scale^100^	Annual (every 6 months for the sleep add-on study subsample)
Adapted version of the 6-item Revised University of California, Los Angeles (UCLA) Loneliness Scale-6^101^	Every 6 months
Difficulties in emotion regulation 16-item version^102 103^	Every 6 months
Cognitive Emotion Regulation Questionnaire^104^	Every 6 months or annual depending on the emotion regulation strategy
Adapted and abbreviated Education Stress Scale for Adolescents^105^	Every 6 months
Alabama Parenting Questionnaire 9-item version^106^	Annual
Abbreviated version of Coping with Adolescent’s Negative Emotions Scale—Adolescent’s Perceptions of Parents^107^	Every 6 months
Peer Relationships Short Form^108^	Every 6 months
Autism Symptom SElf-ReporT for adolescents and adults^109^	Every 2 years
Abbreviated School Climate Measure —abbreviated positive student–teacher relationships subscale^110^	Annual
Romantic relationships measured adapted from Wieczorek *et al*^111^	Every 6 months
Bespoke positive and negative life events questions	Every 6 months
Abbreviated version of the Gratitude Questionnaire 6-item form^112^	Annual
Zurich Brief Bullying Scales victimisation subscale^113^	Annual
Short Digital Activities and Feelings Inventory^114^	Every 6 months

**Table 2:** Parent/caregiver survey measures

Informant reports on child mental health	Frequency
Revised Child Anxiety and Depression Scale^91 92^ 25 item version	Annual
Parental mental health	
Patient Health Questionnaire 9-item version	Annual
Generalized Anxiety Disorder-7^115^	Annual
Abbreviated version of the Perceived Stress Scale^98^	
Parental Stress Scale^116^	Annual
WHO Wellbeing Scale (WHO-5) (WHO, 1998)	Annual
Candidate influences on child mental health	
Social Behavior Questionnaire-Attention Deficit Hyperactivity Disorder (SBQ-ADHD) subscale parent report version^60^	Annual
Coping with Adolescents Negative Emotions Scale—caregiver report^107^	Annual
Cognitive Emotion Regulation Questionnaire^104^	Annual
Alabama Parenting Questionnaire 9-item version^106^	Annual
Closeness and conflict with young person (adapted from the Millennium Cohort Study)^117^	Annual

**Table 3:** Ecological momentary assessment and daily measures

Measure	Frequency
Adapted version of consensus sleep diary^118^	Once per day (morning), every EMA burst
Context (single items for location, company and activity adapted from the D2M study	Four times per day, every EMA burst
Peer interactions (items on inclusion, victimisation and conflict adapted from the Mental Health in the Moment-Attention Deficit Hyperactivity Disorder (MHIM-ADHD) study^64^	Four times per day, every EMA burst
Abbreviated and adapted version of the Positive Affect Negative Affect Scale Child Form (PANAS-C) (one item each for ‘lonely’, ‘happy’, ‘calm’, ‘nervous’, ‘stressed’ and ‘mad’ (angry))	Four times per day, every EMA burst
Social media engagement (bespoke measure based on Digital Activity and Feelings Inventory)^114^	Four times per day, every EMA burst
Abbreviated 5-item version of the Regulating Emotions of Systems Survey EMA (RESS-EMA)^62^	Four times per day, every EMA burst
Romantic relationship experiences (bespoke 4-item measure)	Four times per day, every EMA burst
Anxiety (2 items) and depression (2 items) symptoms^119^	Four times per day, every EMA burst
Adapted and abbreviated version of International Physical Activity Questionnaire^120^	Once per day (evening), every EMA burst
School attendance	Once per day (evening), every EMA burst
Overall assessment of positive feelings	Once per day (evening), every EMA burst
Overall assessment of negative feelings	Once per day (evening), every EMA burst

D2M, Decades-to-minutes; EMA, ecological momentary assessment.

The *six-monthly young person survey* includes information about mental health symptoms and their influences (eg, emotion regulation, academic stress). The *annual caregiver survey* provides complementary information (eg, parental mental health) and a subset of common measures with the young person measures to provide a multi-informant perspective.

The *EMA measures* include information about momentary symptoms and experiences in bursts lasting up to 2 weeks every 6 months. These will be completed by participants on their own smartphones (study smartphones will be provided if participants do not have their own). For each burst, they will be completed up to six times a day (four EMA, one daily diary and one sleep diary) over a 2-week period. Measures of individuals’ affective dynamics will be derived using dynamic structural equation modelling (DSEM). These will include measures such as negative affective levels, lability, inertia and coupling or reactivity (eg, strength of links between stressor and negative affective reaction).^56^

The *ambient sleep measures* will be collected using radar-based technology, which will run continuously for a period that includes the EMA/survey bursts. These data will be collected for a subsample of approximately n=50 of the youngest cohort, to capture a critical period of development for sleep. While polysomnography (PSG) is the gold standard for sleep measurement, it is obtrusive and costly and therefore impractical for measurement burst studies. Recent advances in radar-based technologies allow sleep to be measured with a high degree of accuracy (as validated against PSG) in the course of people’s daily lives.^57^ Data will be used to derive indices of sleep volume and timing. This will be complemented by subjective sleep measures collected as part of the EMA daily diary.

*Hair samples* will be collected at three time-points throughout the study. These will be used to obtain biomarkers of accumulated biological stress over the period concurrent with the EMA period and over the last 6 months.^47 48^ These will be analysed to quantify cortisol and cortisone levels. We are currently piloting the use of nail samples to help us impute hair cortisol/cortisone levels for those unable or unwilling to provide a hair sample. To ensure that possible confounding factors are measured, participants will also complete a set of questions in the *biosampling survey* factors such as medications and hair treatments (eg, dye, bleach, relaxers or hair straighteners) that have been shown in previous work to impact hair cortisol readings.^58 59^

#### Measure selection process

The measure selection was conducted in collaboration with our MHIM-YPAG in a two-stage process. First, young people helped prioritise mental health outcomes and influences (concepts) important to them, then young people helped review and select specific measures for these concepts. Measures that are openly available, that have been shown to possess strong psychometric properties in adolescent samples, that are rated most highly by our MHIM-YPAG and that provide harmonisation and benchmarking opportunities (eg, they have been used in previous nationally representative studies) were prioritised. Where no existing measure is available, measures were adapted or coproduced with our MHIM-YPAG. Further, where there is a clear need for age-tailoring, we will aim to combine sets of developmentally invariant core item sets with age-specific items that capture the manifestation of concepts at different developmental stages.^60^ For example, the Revised Children’s Anxiety and Depression Scale measuring anxiety and depression and the Social Behavior Questionnaire Attention Deficit Hyperactivity Disorder subscale (SBQ-ADHD) measuring ADHD symptoms have versions validated for both children and adults with a core set of items in common,^60 61^ facilitating measurement across the entire developmental span of MHIM.

For the vast majority of survey measures, we were able to adopt measures that had been previously validated in adolescent samples, that were openly available and that were endorsed by our MHIM-YPAG. In a small number of cases, it was necessary to make small wording changes following MHIM-YPAG review in order to address clarity issues or to make the measures more suitable for a UK context. These changes will be clearly documented in publications using the data. Psychometric analyses will be conducted using the data to assess the reliability and validity of all measures as administered in MHIM.

Given that psychometric validation of EMA measures remains uncommon and EMA measures are less well established, the previous evidence pertaining to the validity of our EMA measures is more limited. Some measures came from validated scales, for example, the Regulating Emotion Systems Survey EMA (RESS-EMA),^62^ some came from previous studies where they had been used successfully but not explicitly psychometrically validated,^63 64^ and others were developed specifically for this study. Psychometric evaluation of these items will be conducted and reported using the data collected.

Minimising burden was also considered during measure selection. We aimed to include a balance of both positive and negative measures, to use short versions of questionnaires where available and where it would not result in a loss of important content and to cap the estimated completion time at approximately 30 min for surveys and 2 min for EMA, in line with MHIM-YPAG views. Further, the measures will be presented in a visually appealing manner and interspersed with interesting facts related to mental health and the brain.

### Statistical analysis

Data will be analysed using a range of approaches depending on the specific research question. A majority of research questions will be addressed using DSEM that can account for the multilevel, time-series (in the EMA) and longitudinal nature of the data.^65^ This can be extended to a measurement burst design to, for example, analyse the cross-lagged relations between day-to-day dynamics and long-term development.^66^ Longitudinal analyses will depend on the assumptions of cohort and longitudinal measurement invariance,^67^ which will be tested prior to the main analyses. Missing data will primarily be dealt with using full information maximum likelihood estimation (FIML), Bayesian estimation, attrition weighting or multiple imputation, depending on the analysis, all of which provide unbiased estimates under an assumption of missing at random.^68^ Robust estimators, such as robust maximum likelihood estimation, will be used to deal with violations of maximum likelihood estimator assumptions.

#### Complex survey design

Non-representativeness of the sample with respect to the underlying national population will be examined and may be addressed by deriving weights for each respondent based on key demographic data collected in comparison with the latest available census summary data.^69^ This and clustering by schools will be accounted for in statistical analyses using standard methods for analysing complex survey data, such as pseudomaximum likelihood estimation or replication weights.^70^

#### Sample size

The sample size was determined based on previous Monte Carlo simulations of DSEMs, which will represent our primary analysis approach. These have suggested that 50 respondents with at least 10 measurement points are needed to avoid small sample bias for random effects, as well as to provide adequate coverage for within-person and between-person effect sizes of a magnitude that are likely to be of clinical importance.^71 72^ We also conducted our own Monte Carlo simulations that used realistic parameters from previous EMA models, which confirmed that a sample size of 50 should be adequate. However, given the likelihood of attrition over time and of missingness within each burst, we will aim for a minimum of 100 respondents per cohort (with the goal of over-recruiting by up to 50% at baseline to allow for attrition) to help ensure an adequate sample size for within-cohort analyses. Specific Monte Carlo analyses may be conducted to assess the extent to which our realised sample size can support particular analyses.

To help maximise retention, we have codesigned several strategies with a YPAG and will continue to refine these strategies with our current MHIM-YPAG.^73^ These strategies were based on a narrative review^74^ and can be organised into four domains: (1) using a codesign approach to ensure the study is appealing for adolescents (eg, consult the YPAG on the study contents to ensure it addresses questions that are relevant for them,^75^ (2) rapport/relationship building with schools and participants (eg, select and train staff for ability to build rapport with participating schools/adolescents; visit schools to present the project and recruit students within each school to act as ‘project ambassadors’), (3) offering reimbursement and incentives (eg, offering shopping vouchers to participants, embedding ‘mental health facts’ in the data collection process, offering school-level feedback) and (4) minimising barriers and burden (eg, using tried and tested technology to minimise glitches, avoiding exam times, not requiring caregiver participation as an inclusion criterion for adolescent participation).

#### Piloting

The main data collection was preceded by a pilot study which aimed to test the core elements of the protocol over two bursts of data collection, lasting 2 weeks each, with a 1-month period between them. The pilot recruited n=68 adolescents to a study, which implemented a single round of survey/EMA data collection and approximately 50% also completed a second round of data collection. This allowed us to test the majority of the elements of the protocol, gain feedback from participants and identify refinements. Members of the MHIM-YPAG will also complete this protocol and provide in-depth feedback.

#### Ethics and dissemination

The study received ethical approval from the philosophy, psychology and language science ethics committee at the University of Edinburgh (04-2425/3). Caregiver informed consent (if young person is under 16) and adolescent assent will be obtained prior to data collection and signposting to mental health support is provided to participants at various points. The study will be delivered in collaboration with schools for which local authority approval will first be obtained. Participants will be clearly informed that the study addresses topics that they may find sensitive. They will also be told that they can skip questions and will not have to view questions on suicidal ideation if they choose not to.

Results will be disseminated via peer-reviewed publications and presentations, and data will be documented and made openly available via repositories and platforms such as the UK Data Service and Open Science Framework.

## Discussion

Motivated by a need for better information on how to support adolescent mental health, MHIM takes a multitimeframe developmental perspective and examines the links between day-to-day experiences and longer-term mental health development. In doing so, it seeks to illuminate how the daily life experiences of adolescents can impact their mental health in the short term and long term and identify and prioritise intervention targets in this key period of development.

MHIM will leverage the (complementary) advantages of EMA, biosampling and traditional survey data. For example, EMA provides rich, high temporal resolution data on mental health dynamics and influences in an ecological context; biosample data can provide objective markers of stress (and hair samples in particular are minimally invasive); and online survey data can gather comprehensive information efficiently. It will draw on both caregiver and young person self-report surveys to provide a multi-informant perspective. It will use an accelerated cohort design to facilitate developmental analyses sooner in the project lifecycle compared with a traditional cohort design and a measurement burst design to capture the connections between daily life experiences and long-term development.

A key set of insights that can be gained from MHIM includes improved multitimeframe developmental theories that explicitly address the links between daily life experiences (cognition, affect, behaviours, events) and long-term mental health development in adolescence. While it is widely acknowledged that cumulative experiences shape mental health, challenges in sustaining high temporal resolution data over longer-term ‘developmental’ time mean that there is a lack of direct empirical data on these links.^30^

A wide range of multitimeframe research questions can be addressed with the data that will be generated. These include those relating to the effects of cumulative daily life experiences on stress and mental health, developmental changes in daily life emotion dynamics and influences on mental health, and reciprocal relations between mental health development and daily life experiences. The data generated from the project will be made openly available to ensure that the data can be used to address as wide a range of research questions as possible, following Findable, Accessible, Interoperable and Reusable principles.^76^

‘Momentary’ influences on and daily life dynamics of symptoms are also increasingly being recognised as valuable for real-time prediction and intervention targeting, via, for example, just-in-time adaptive interventions and other digital health interventions.^35 77^ Such interventions are seen as promising, particularly given that young people may be reluctant to seek help or have difficulties accessing services.^78^ Digital interventions such as smartphone-based applications, in contrast, are widely accessible.^79^

### Limitations

There are, however, important limitations of the design that should be acknowledged. First, the intensive and long-term data collection schedule means that retention will be a significant challenge. The engagement protocol for the project^73^ informed by young person consultations and the existing evidence on effective engagement of adolescents in research^74^ is designed to mitigate these challenges and includes strategies relating to coproduction, relationship building, burden/barrier reduction and incentives. To mitigate the impacts of missing data, a very short survey will be issued to those who do not respond to the invitation to participate in the standard protocol. However, some (non-random) attrition and missing data is inevitable. This will reduce the available observations and potentially bias parameter estimates if not suitably adjusted for.^80^ To ensure that the burden is not excessive, there will be limits on the comprehensiveness of the data that will be collected. There are consequently many concepts relevant for adolescent mental health that will inevitably not be possible to measure.^81^ Our focus will be on mental health influences that are prioritised by adolescents themselves, and that may represent the most proximal and malleable intervention targets. Thus, although it is acknowledged that symptoms in this period may have origins much earlier in development, as far back as the prenatal period,^82^ these earlier influences will not generally be within the scope of the present study.

Engagement challenges are also likely to manifest in compliance with the EMA protocol. Previous research suggests that compliance rates can vary substantially and may be somewhat related to respondent characteristics and momentary states, potentially creating biases in the data which are only partially addressed by missing data methods such as Bayesian estimation, FIML estimation, multiple imputation and weighting.^3483^,^85^ In UK-based adolescent populations, compliance is made more challenging by policies that limit smartphone use during school hours. Taken together, in the current study based on previous similar research, we may expect to see average compliance rates around 60%–70%^83 85^; and may particularly miss information about an important context (school) in which daily experiences shape mental health.

Owing to the observational nature of the study, we will rely on statistical adjustment for confounding through, for example, counterfactual methods when exploring causal hypotheses related to mental health influences.^86 87^ This is naturally likely to be more vulnerable to violations of the assumption of independence of treatment assignment and potential outcomes as compared with randomised designs. The repeated measures design will allow us to exploit methods in which young people act, in a sense, as their own controls and we can examine the impacts of variations in mental health predictors on mental health outcomes. This will, however, be vulnerable to unmeasured confounding by time-varying confounders.

Given the intensive data collection design, there may be measurement reactivity occurring within the design (ie, where participants’ behaviour changes as a result of participation in the study). The possibility of measurement reactivity within EMA and measurement burst studies is understudied. By examining measurement changes over time,^88 89^ our study can contribute insights into the extent to which it occurs and may impact inferences drawn from EMA and measurement burst studies. We will also leverage the accelerated cohort design to explore the extent to which, at each age, those who have been in the study longer show different response patterns (eg, comparing those who were 15 at baseline vs in years 2, 3 and 4 of the study).

Finally, many of the EMA measures do not have established psychometric properties in adolescents, reflecting the relatively nascent state of EMA research compared with traditional survey research, coupled with a lesser focus on psychometric testing.^90^ Together, this means that many EMA measures are not yet psychometrically validated or have to be newly developed.
