# Supplementary material for: Mental health in the moment: protocol for an accelerated cohort measurement burst study of adolescent mental health
Source: BMJ Open. 2025 Nov 12;15(11):e110190. doi: 10.1136/bmjopen-2025-110190 (PMC12612733; doi:10.1136/bmjopen-2025-110190)
Supplement: Supplementary file 1 [file bmjopen-2025-110190.reviewer_comments.pdf]

PEER REVIEW HISTORY

BMJ Open publishes all reviews undertaken for accepted manuscripts. Reviewers are asked to complete a checklist review form and are provided with free text boxes to elaborate on their assessment. These free text comments are reproduced below.

ARTICLE DETAILS

Title (Provisional)

Mental health in the moment: Protocol for an accelerated cohort measurement burst study of adolescent mental health

Authors

Murray, Aja; Power, Luke; Hoxha, Dejla; Xie, Tong; Wright, Helen; Caddick, Lorna; Dryburgh, Katie; Sanchez-Izquierdo, Clara; Melashenko, Daria; Crocker, Alex

---

This article was not externally reviewed at BMJ Open. Protocols that have been independently assessed prior to submission to BMJ Open are usually fast-tracked to publication on the grounds that further substantial changes will not be possible. This independent assessment will usually be external, independent review for both a substantial grant award from a non-commercial or government funder and ethics approval.
